# Algorithmic fandom in the age of AI: a four-wave multilevel longitudinal study of identity mirroring, fan belonging, and social inclusion

**DOI:** 10.3389/fpsyg.2026.1955806

**Published:** 2026-10-19

**Authors:** Haibo Wang

**Affiliations:** School of Physical Education Institute, Hechi University, Hechi, China

**Keywords:** algorithmic identity mirroring, digital fan communities, digital identity integration, fan belonging, participatory algorithmic governance, social inclusion

## Abstract

**Introduction:**

AI-curated sports environments can make users feel that a platform recognizes who they are, yet the psychological process linking perceived recognition to belonging and inclusion remains unclear.

**Methods:**

We examined this process in a four-wave, multilevel longitudinal survey of 1,200 participants nested within 60 digital sports communities in China. Participatory algorithmic governance was examined as a community-level boundary condition. Community fixed-effects models with standard errors clustered by community were used, with prior levels of the corresponding outcomes and participant characteristics taken into account. The serial prospective association at mean governance climate was evaluated using a community bootstrap with 2,000 replications.

**Results:**

Algorithmic identity mirroring at Wave 1 was positively associated with digital identity integration at Wave 2, digital identity integration was positively associated with fan belonging at Wave 3, and fan belonging was positively associated with perceived social inclusion at Wave 4. The models supported all three lagged associations, and the community bootstrap supported a positive serial prospective association at mean governance climate. Participatory governance modestly strengthened the association between algorithmic identity mirroring and subsequent digital identity integration but did not attenuate the negative association between marginalized identity and later perceived social inclusion.

**Discussion:**

Conceptually, the study distinguishes algorithmic identity mirroring from personalization, algorithmic trust, and self-congruity, while also differentiating digital identity integration, fan belonging, perceived social inclusion, and team identification. The findings provide longitudinal evidence consistent with the proposed psychological sequence, although significant reverse-order diagnostics indicate that reciprocal associations remain plausible. Because the study is observational, the findings should be interpreted as longitudinal associations rather than causal effects.

## 1 Introduction

AI-curated social environments increasingly organize the cues through which people encounter, interpret, and express identity. Recommendation algorithms, personalization systems, creator rankings, highlight feeds, and communities mediated by AI do more than deliver information. They influence which histories, affiliations, rituals, styles, and group signals become visible and recurrent within a digital environment. In fan communities, these selections can make a platform appear to recognize a user as a particular kind of fan, giving algorithmic curation psychological significance beyond the ranking of content (Gillespie, 2014). Yet receiving personally relevant content is not equivalent to feeling recognized by the system that selected it. Existing research on human interaction with AI and personalization has largely examined trust, acceptance, usability, satisfaction, transparency, and decision outcomes. These perspectives explain important dimensions of how people evaluate algorithmic systems (Sundar and Marathe, 2010; Tam and Ho, 2006; Shin, 2020), but they do not fully explain when users interpret curated outputs as reflections of who they are. A feed can be relevant without becoming identity relevant, and a system can be trusted without making a user feel recognized. Research on algorithmic imaginaries and folk theories further suggests that people respond not only to what computational systems do, but also to what they believe those systems notice and value about them, even when the underlying operations remain opaque (Bucher, 2017; DeVito et al., 2018; Rader and Gray, 2015; Eslami et al., 2015; Cotter, 2019). The central psychological question is therefore not whether an AI system objectively knows a person, but whether users interpret its outputs as evidence that the system recognizes who they are. Users encounter patterns of recommendation, repetition, omission, and surprise and infer from these patterns what the system appears to understand about them. When curated content repeatedly foregrounds a team history, athlete style, fan ritual, or community discourse that carries personal meaning, perceived relevance may become perceived recognition. We refer to this experience as algorithmic identity mirroring, defined as the perception that outputs curated by AI reflect meaningful aspects of one’s identity, preferences, values, and group affiliations. Algorithmic identity mirroring therefore concerns a psychological interpretation of algorithmic curation rather than the objective accuracy of a recommendation system. It identifies a theoretically distinct point at which algorithmic attention may become identity-relevant information.

Perceived recognition, however, becomes psychologically consequential only if it is incorporated into a broader process of self-organization and social confirmation. We propose a sequential framework in which algorithmic identity mirroring provides symbolic material that may contribute to digital identity integration, which concerns whether experiences of fandom mediated through a platform become coherent with the wider digital self. This distinction separates the experience of being recognized from the subsequent organization of that recognition into a meaningful self-concept. Self-concept, narrative identity, and social identity perspectives each suggest that experiences acquire psychological weight when they are organized into a coherent and socially anchored self (Ashforth and Mael, 1989; Ellemers et al., 2002). From this perspective, curated fan signals can provide identity material without necessarily becoming part of the self. Integration represents the psychological transition through which such material may acquire coherence within a person’s digital identity. Yet coherence within the self is still distinct from confirmation by others. Fan belonging captures this next transition by referring to felt acceptance, membership, and relational embeddedness within a focal fan community. Perceived social inclusion, in turn, concerns whether such relational confirmation is accompanied by perceived access to valued participation, recognition, and social relations within digital fan communities. The proposed sequence therefore distinguishes four related but noninterchangeable processes: perceived recognition, self-organization, relational confirmation, and social inclusion. Importantly, these processes do not unfold in an institutional vacuum. The meaning assigned to algorithmic recognition may depend on whether community members experience rules governing curation as intelligible, contestable, and responsive to their voice. Participatory algorithmic governance captures this shared community context through opportunities to understand recommendation practices, provide feedback, contest errors, and encounter representation in procedures governing curation. Such participation may make algorithmic recognition appear more answerable and legitimate, thereby strengthening the association between perceived mirroring and identity integration. Procedural legitimacy, however, should not be equated with substantive inclusion. Opportunities for voice, feedback, and appeal do not necessarily eliminate identity-related disadvantage or guarantee equal access to valued participation. The framework therefore distinguishes the conditions under which governance may reinforce the psychological meaning of algorithmic recognition from the stronger claim that participatory procedures can remedy marginalization.

The present study develops and evaluates this framework using a four-wave, multilevel longitudinal survey of participants engaged in digital sports communities in China. It makes three related contributions. First, it develops algorithmic identity mirroring as a psychological construct that captures perceived recognition by an intelligent platform environment and distinguishes this experience conceptually from personalization, algorithmic trust, and self-congruity. Second, it specifies a sequential account in which perceived algorithmic recognition is associated with digital identity integration, subsequent fan belonging, and later perceived social inclusion. This formulation distinguishes digital identity integration, fan belonging, perceived social inclusion, and team identification rather than treating them as interchangeable expressions of attachment to a sports community. Third, it places this psychological sequence within a community governance context by examining whether participatory algorithmic governance conditions the association between perceived recognition and identity integration and whether it attenuates identity-related disparities in perceived social inclusion. Methodologically, the four-wave design provides temporal separation among the focal constructs, while the multilevel structure allows individual experiences to be examined in relation to governance conditions shared within digital sports communities. These features permit a temporally ordered assessment of the proposed associations and their community-level boundary conditions.

## 2 Theory and hypotheses

### 2.1 Algorithmic identity mirroring: AI-mediated recognition of the self

Algorithmic identity mirroring refers to the extent to which individuals perceive outputs curated by AI as reflecting meaningful aspects of their identity, preferences, values, and group affiliations. The construct is psychological rather than technical, capturing the subjective experience of being recognized rather than the objective accuracy with which a recommender infers preferences or predicts behavior. An algorithmic system can therefore be accurate, useful, or engaging without being experienced as a reflection of the self. Mirroring emerges when users interpret patterns of platform selection as identity-relevant signals and infer that the intelligent environment appears to understand something meaningful about who they are. Such perceived recognition may provide symbolic material for digital identity integration. Teams, athlete styles, rituals, histories, and community narratives can become more than recommended content when their correspondence with personally meaningful aspects of fandom allows users to connect platform experiences with the broader digital self. Research on self-concept clarity emphasizes a clearly defined and internally consistent sense of self, narrative identity research explains how experiences are organized into an evolving self-story, and social identity theory explains how group memberships become incorporated into self-definition (Tajfel and Turner, 1979; Campbell et al., 1996; McAdams, 2001; Brewer, 1991; Cruwys et al., 2014). Digital identity integration extends this logic to environments mediated by AI by asking whether curated fan signals become coherent components of the wider digital self rather than remaining disconnected traces of platform activity.

Algorithmic identity mirroring and digital identity integration therefore represent distinct constructs within the proposed sequence. Mirroring concerns perceived recognition, whereas integration concerns the organization of identity-relevant cues within a coherent digital self. This distinction also separates mirroring from adjacent concepts. Relevant content may attract attention without becoming identity material, trust in an algorithmic system does not necessarily imply coherence within the digital self, and an object or brand may correspond with a user’s self-image without producing the perception that an intelligent system has recognized and reflected that identity. Algorithmic identity mirroring is thus distinct from content relevance, algorithmic trust, engagement, and self-congruity, while digital identity integration captures the extent to which identity-relevant platform experiences become incorporated into the digital self. Table 1 summarizes these conceptual boundaries. On this basis, we propose the following prospective association:

**Table 1:** Conceptual boundary of algorithmic identity mirroring.

Construct	Core question	Primary object	Boundary from AIM
Personalization	Is the content relevant?	Fit between content and preference	AIM asks whether relevance is interpreted as identity recognition.
Algorithmic trust	Can I rely on the system?	Reliability, fairness, or competence	AIM concerns perceived recognition, not reliance on outputs.
Self-congruity	Does the object match my self-image?	Fit between self-image and object or brand	AIM emphasizes an intelligent system reflecting identity back to the user.
Algorithmic identity mirroring	Does the system appear to understand who I am?	AI-curated identity cues and affiliations	AIM is the perceived recognition event that may precede identity integration.

AIM, algorithmic identity mirroring.

*H1*: Higher perceived algorithmic identity mirroring at Wave 1 will be positively associated with higher digital identity integration at Wave 2, controlling for prior integration and participant characteristics.

### 2.2 Identity integration, fan belonging, and inclusion

Fan belonging refers to felt acceptance, membership, and relational embeddedness within a focal fan community (Baumeister and Leary, 1995) and may provide relational confirmation of an integrated fan identity. It is distinct from private team identification (Heere and James, 2007; Lock and Heere, 2017), consumption frequency, behavioral engagement, or parasocial attachment to an athlete. Research on psychological sense of community identifies membership, influence, shared emotional connection, and need fulfillment as distinct dimensions of collective experience (McMillan and Chavis, 1986), while research on virtual communities shows that such experiences can emerge in digital settings without being reduced to message volume or platform use (Blanchard, 2007). Digital identity integration may facilitate this relational process because a coherent fan identity can help users present a recognizable sense of self, identify compatible community practices, and interpret social interaction as affirming that identity. Integration and belonging therefore capture different psychological processes. The former concerns coherence within the digital self, whereas the latter concerns acceptance, membership, and relational connection within a community.

Belonging, in turn, is conceptually distinct from perceived social inclusion. Relational membership can provide reciprocal recognition, opportunities for participation, and social ties that connect platform experiences with valued relationships. A sociometer account proposes that self-relevant evaluations are responsive to relational acceptance, making belonging a plausible bridge between identity expression and perceived inclusion (Leary et al., 1995). Perceived social inclusion nevertheless extends beyond the feeling of membership by capturing perceived access to valued participation, recognition, and social relations within digital fan communities. The proposed framework is deliberately bounded. It does not imply that AI eliminates social inequality or produces inclusion in society more generally. Rather, it specifies a prospective sequence in which digital identity integration is expected to be associated with subsequent fan belonging, which is in turn expected to be associated with later perceived social inclusion within particular digital fan communities. Sports communities provide a useful context for examining this sequence because symbolic affiliation is visible, emotionally salient, and organized around shared group membership. Table 2 clarifies the boundaries among these constructs and adjacent concepts. Given the temporal separation of the focal constructs and adjustment for prior levels of the corresponding outcomes, the ordered product is interpreted as a serial prospective association rather than a causally identified mediation effect. The hypothesized temporal ordering does not imply an exclusive direction; because belonging and inclusion may in turn reinforce identity integration and the perception of being recognized, reciprocal associations are also plausible and are examined in the diagnostic analyses. We therefore propose:

**Table 2:** Distinguishing identity integration, fan belonging, social inclusion, and adjacent constructs.

Construct	Defining focus	What it is not	Role in the model
Digital identity integration	Coherence between platform-mediated fandom and the wider digital self	A feed match, self-presentation tactic, or strength of team attachment	Self-organization after perceived recognition
Fan belonging	Felt acceptance, membership, and relational embeddedness in a focal fan community	Private team identification or behavioral engagement alone	Relational confirmation of an integrated fan self
Perceived social inclusion	Perceived access to valued participation, recognition, and social relations within digital fan communities	General societal inclusion or emotional membership alone	Bounded downstream social outcome
Team identification	Psychological connection between a person and a sports team	Community acceptance or access to participation	Adjacent sport-identity construct, not a mediator in the focal sequence
Psychological sense of community	Broad membership, influence, need fulfillment, and shared emotional connection	A platform-specific identity integration process	Broader community framework overlapping most with belonging

Construct boundaries follow their roles in the proposed sequence; no row implies that adjacent concepts are unrelated.

*H2*: Higher digital identity integration at Wave 2 will be positively associated with higher fan belonging at Wave 3, controlling for prior belonging and participant characteristics.

*H3*: Higher fan belonging at Wave 3 will be positively associated with higher perceived social inclusion within digital fan communities at Wave 4, controlling for prior inclusion and participant characteristics.

*H4*: The ordered product linking algorithmic identity mirroring, digital identity integration, fan belonging, and perceived social inclusion within digital fan communities will be positive.

### 2.3 Participatory governance and marginalized-identity boundaries

Participatory algorithmic governance climate refers to a shared perception that community members have meaningful voice, accessible feedback and appeal channels, and representation in rules governing algorithmic curation. It is conceptualized at the community level because governance becomes socially consequential when members experience these arrangements as a common institutional property rather than as isolated individual encounters. The construct is therefore distinct from general trust in an algorithmic system or satisfaction with a single complaint outcome. Research on the governance of common resources suggests that durable institutions make rules intelligible and provide affected members with opportunities to participate in their modification (Ostrom, 1990). Applied to digital platforms, these principles suggest that participatory governance may make algorithmic identity cues appear more accountable and responsive to the community (Helberger et al., 2018). Such a climate may strengthen the prospective association between algorithmic identity mirroring and digital identity integration because voice and contestability can influence how users interpret identity-relevant content. Under more participatory conditions, correspondence between curated content and identity may be interpreted as recognition within a system that can be questioned and revised. Under less participatory conditions, similar correspondence may appear accidental, opaque, or manipulative.

The implications of participatory governance for marginalized identity involve a stronger and conceptually distinct claim. Voice, contestability, and representation may support more responsive participation, but procedural legitimacy does not necessarily produce substantive inclusion. Scholarship on racialized technology cautions that improved interfaces and procedures do not by themselves displace institutional patterns embedded in data and design priorities (Benjamin, 2019; Kellogg et al., 2020). Participants who identify as marginalized are therefore expected to report lower perceived social inclusion; this disparity is examined as an expected association rather than as a numbered hypothesis, and it provides the baseline on which H6 builds. Participatory governance may therefore strengthen the psychological significance of algorithmic recognition without necessarily reducing identity-related disparities in perceived social inclusion. These moderation propositions are related but not logically interchangeable, and they operate across distinct levels of analysis. Participatory algorithmic governance is conceptualized as a community-level climate derived from member assessments, whereas algorithmic identity mirroring and marginalized identity are individual-level variables. The first interaction examines whether community governance conditions the prospective association between mirroring and subsequent integration. The second examines whether governance attenuates the expected negative association between marginalized identity and later perceived social inclusion. This distinction allows the model to examine both the potential legitimizing role and the substantive limits of participatory governance. We therefore propose:

*H5*: Participatory algorithmic governance climate positively moderates the association between Wave 1 algorithmic identity mirroring and Wave 2 digital identity integration.

*H6*: Participatory algorithmic governance climate attenuates the negative association between marginalized identity and Wave 4 perceived social inclusion.

### 2.4 AI as an identity-relevant social environment

The framework treats AI neither as a neutral delivery mechanism nor as a human social partner, but as an identity-relevant social environment that selects, ranks, and repeats cultural cues that users may interpret as recognition, misrecognition, or constraint. This perspective shifts attention from whether users simply accept or trust AI to how they assign psychological meaning to identity cues curated by intelligent systems. Within digital fan communities, these interpretations may shape how platform experiences become organized within the self and socially confirmed. The framework remains bounded to perceived inclusion within a focal digital community and does not imply the elimination of identity-related or structural disadvantage (Figure 1).

**Figure 1:**
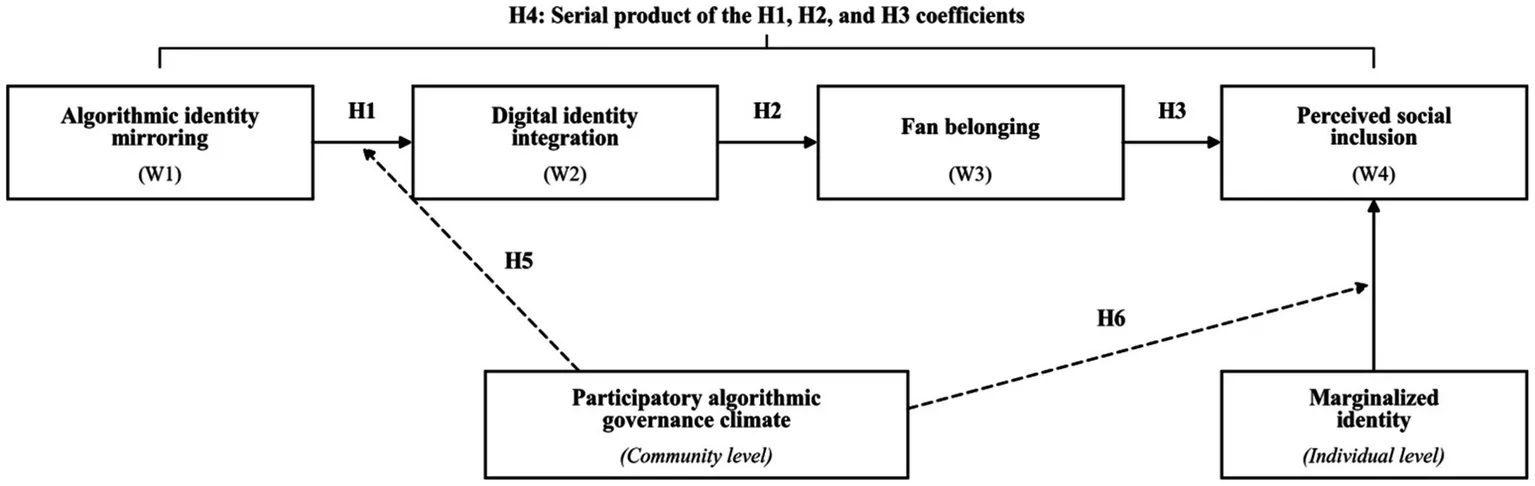
Four-wave cross-level conceptual model and hypotheses. W, wave. Prior levels of each dependent variable, participant characteristics, and community fixed effects are included in the corresponding equations but omitted from the figure for readability. Solid arrows denote proposed prospective associations, and dashed arrows denote cross-level moderation; H4 is the product of the H1, H2, and H3 coefficients. The association between marginalized identity and Wave 4 perceived social inclusion is an expected association rather than a numbered hypothesis.

## 3 Methods

### 3.1 Study design, context, and recruitment

This study used a four-wave longitudinal survey design (Little, 2013) conducted in China to examine prospective associations among algorithmic identity mirroring, digital identity integration, fan belonging, and perceived social inclusion within digital sports communities. Data were collected through Wenjuanxing, an online survey platform widely used for questionnaire administration in China. Participants were recruited through multiple channels associated with digital sports engagement, including WeChat groups, QQ groups, sports fan communities, educational settings, and social media. This recruitment strategy was designed to reach individuals participating in different forms of digitally mediated sports communities rather than relying on a single recruitment source. The baseline sample comprised 1,200 participants nested within 60 digital sports communities. The digital sports platform contexts represented in the study are summarized in Table 3.

**Table 3:** Digital sports platform contexts represented in the study.

Platform context	Archetype	Use in this study
NBA App/NBA digital community	League-centered content and fandom	Digital sports platform context
ESPN	Sports media and personalized sports content	Digital sports platform context
Nike App/Nike digital community	Brand and activity community	Digital sports platform context
adidas App/adidas digital community	Brand and sport lifestyle community	Digital sports platform context
Strava	Activity network and peer community	Digital sports platform context

These platforms represent digital sports environments used by participants and provide the empirical context for interpreting platform-related fandom and algorithmic curation. They were not survey administration platforms or recruitment channels. Survey data were collected through Wenjuanxing, while participants were recruited through WeChat groups, QQ groups, sports fan communities, educational settings, and social media.

Data were collected across four temporally separated measurement occasions, with approximately 8 weeks between successive waves. All five focal constructs were assessed at every wave. The focal equations used algorithmic identity mirroring from Wave 1, digital identity integration from Wave 2, fan belonging from Wave 3, and perceived social inclusion from Wave 4, with the prior-wave measure of each outcome retained for longitudinal adjustment. The design therefore established temporal separation between each focal predictor and its outcome, and the repeated measurement of every construct also permitted the alternative temporal ordering checks reported in Section 4.9.

### 3.2 Panel structure and participant characteristics

The baseline sample comprised 1,200 participants distributed across 60 digital sports communities. Participant characteristics included age in years, tenure in the focal digital sports community in months, a woman indicator, and a marginalized identity indicator. The marginalized identity variable was measured at the individual level and was included to examine whether participants reporting marginalized identities differed in subsequent perceived social inclusion. Because this variable captured broad self-identified marginalization rather than membership in specific identity categories, its questionnaire wording, illustrative examples, and coding procedure are described in Section 3.3 and Supplementary Table S1 so that the construct is interpreted within the scope of what was actually assessed.

The longitudinal panel included 1,200 participants at Wave 1, 1,147 at Wave 2, 1,090 at Wave 3, and 1,012 at Wave 4, corresponding to retention rates of 95.6%, 90.8%, and 84.3% relative to the baseline sample. Missing data arose from participant attrition across waves and item nonresponse within measurement occasions. Scale scores were calculated when at least three of the four corresponding items were available. A complete-item sensitivity analysis for H1 was conducted to assess whether the primary findings were materially affected by the observed missingness pattern.

### 3.3 Constructs, indicator domains, and expert review

Five focal constructs were assessed using four items each, with responses recorded on a seven-point scale. Algorithmic identity mirroring (AIM) assessed the extent to which participants perceived curated content as reflecting meaningful aspects of their identity, preferences, values, and group affiliations. Digital identity integration (DII) assessed the extent to which fandom experienced through digital platforms was coherently incorporated into the broader digital self. Fan belonging (FB) assessed perceived acceptance, membership, and relational connection within the focal fan community. Perceived social inclusion (SI) assessed perceived access to valued participation, recognition, and social relations within digital fan communities. Participatory algorithmic governance (PG) assessed perceived voice, feedback opportunities, appeal mechanisms, and representation in rules governing algorithmic curation. The fourth item of each construct was negatively keyed and reverse scored as 8 minus the original response before scale scores were calculated.

Marginalized identity was assessed separately from the five focal constructs using a binary self-identification item asking participants whether they considered themselves to belong to a marginalized or socially disadvantaged identity group. The questionnaire provided illustrative examples of potentially marginalized identities to clarify the meaning of the item and assist participants in making this judgment, rather than requiring them to identify with or select a specific identity category. Participants who responded yes were coded 1, and those who responded no were coded 0. At baseline, 407 participants, representing 33.9% of the sample, were coded as reporting a marginalized identity. Because the measure captures broad self-identified marginalization rather than membership in separately measured identity categories, its association is interpreted at the aggregate level and should not be interpreted as an estimate for any particular social group. The original Chinese wording, illustrative examples, English translation, response options, and coding procedure are reported in Supplementary Table S1.

The construct definitions and item content were reviewed by two experts before data collection. Both experts judged the proposed content to be consistent with the intended conceptual domains, and no changes were made following their review. To provide full transparency for the newly operationalized constructs and facilitate future replication, Supplementary Table S1 reports all 20 focal construct items as administered, together with the marginalized identity item, original Chinese wording, English translations, response options, construct assignments where applicable, and scoring or coding directions. Evidence concerning internal consistency, convergent validity, discriminant validity, and the factor structure of the focal measures is reported in the subsequent measurement assessment.

### 3.4 Measurement and aggregation assessment

Measurement analyses were conducted in R version 4.5.3 using lavaan version 0.6–21. The seven-point item responses were treated as continuous, and the negatively keyed fourth item of each construct was reverse scored before analysis. Separate confirmatory factor analyses were estimated at each wave using robust maximum likelihood (MLR) and cases with complete data on all 20 focal items for that wave. The hypothesized model specified five correlated factors. All cross-loadings and residual correlations were fixed to zero. Model fit was evaluated using the robust comparative fit index (CFI), Tucker–Lewis index (TLI), root mean square error of approximation (RMSEA) with its 90% confidence interval, and standardized root mean square residual (SRMR). The five-factor solution was compared with models that combined AIM with DII, combined FB with SI, or assigned all items to a single factor. Internal consistency was assessed using coefficient alpha (Sijtsma, 2009) and composite reliability (Raykov, 1997) derived from standardized CFA loadings. Convergent validity was evaluated using average variance extracted, while discriminant validity was assessed using heterotrait–monotrait ratios, with values below 0.85 taken as evidence of discriminant validity (Henseler et al., 2015). Longitudinal measurement invariance was not evaluated because an invariance testing sequence was not prespecified (Putnick and Bornstein, 2016).

Participatory algorithmic governance (PG) was aggregated from individual assessments at Wave 1 after examining whether the data supported its representation as a shared community-level climate. Aggregation was evaluated using the intraclass correlation coefficients, ICC(1) and ICC(2) (Shrout and Fleiss, 1979), together with median agreement within communities (LeBreton and Senter, 2008). An average of 19.95 valid participant responses contributed to each community estimate. Each community mean was assigned to all baseline participants in that community. For the primary interaction models, these assigned values were standardized across the 1,200 baseline participant records using their mean (4.007) and sample standard deviation (0.720; *N* − 1 denominator). Thus, PGz = 0 corresponds to the mean governance climate. This scaling is distinct from the sample standard deviation across the 60 unique community means (0.726). These diagnostics were used to determine whether sufficient between-community variation and within-community agreement were present to support aggregation of individual PG assessments to the community level.

### 3.5 Statistical specification

Three prespecified lagged equations estimated digital identity integration (DII) at Wave 2 from algorithmic identity mirroring (AIM) at Wave 1, fan belonging (FB) at Wave 3 from DII at Wave 2, and perceived social inclusion (SI) at Wave 4 from FB at Wave 3. Each equation adjusted for the prior-wave level of the corresponding dependent variable, age, the woman indicator, marginalized identity, and community fixed effects (Mundlak, 1978). In the DII equation, individual AIM was centered within community (Enders and Tofighi, 2007), and the model additionally included the interaction between AIM and standardized community participatory algorithmic governance (PG) (Preacher et al., 2007). The SI equation included the interaction between marginalized identity and PG (Hayes, 2018). Because PG is constant within each community, its main effect is absorbed by the community fixed effects and cannot be estimated separately. The interaction terms remain identified because the individual-level predictors vary within communities. This specification therefore separates within-community individual variation from time-invariant differences across communities while allowing the associations between individual-level predictors and subsequent outcomes to vary according to community PG. The AIM coefficient in the DII equation and the marginalized-identity coefficient in the SI equation are conditional associations evaluated at PGz = 0. In these primary equations, participant characteristics refer to age, the woman indicator, and marginalized identity; community tenure was reported descriptively (Table 4) and was used only in the separate predictive comparison described below.

**Table 4:** Panel profile and missingness.

Characteristic	Value
Baseline participants	1,200
Digital sports communities	60
Participants at Wave 2	1,147
Participants at Wave 3	1,090
Participants at Wave 4	1,012
Marginalized identity indicator	407 (33.9%)
Woman indicator	569 (47.4%)
Age, mean years	34.33
Tenure, median months	24.0
Missing item responses	8,724

Participant characteristics are reported for the baseline sample. Missing item responses reflect participant attrition across waves and item nonresponse within measurement occasions. Wave refers to the corresponding measurement occasion.

Standard errors were clustered by community. Given the finite number of 60 community clusters, hypothesis tests and 95% confidence intervals were based on a t distribution with 59 degrees of freedom rather than on large-sample normal approximations. H1, H2, H3, and H5 were considered supported when the corresponding coefficient was in the hypothesized direction with *p* < 0.05; H6 was considered supported when the marginalized identity × PGz interaction was positive with *p* < 0.05; and H4 was considered supported when the 95% bootstrap confidence interval for the ordered product excluded zero. For H4, the serial statistic was defined as the product of the H1, H2, and H3 coefficients from the primary equations described above, retaining the AIM × PGz interaction in the DII equation and the marginalized identity × PGz interaction in the SI equation. Because the H1 coefficient is evaluated at PGz = 0, the reported product represents the ordered association at mean governance climate; it is not a governance-invariant product. Uncertainty in this product was estimated using 2,000 community-level bootstrap replications, thereby preserving the nested structure of the data. Diagnostic analyses included models with alternative temporal ordering and a complete four-item sensitivity analysis for H1 that required complete responses for AIM at Wave 1 and DII at Waves 1 and 2. These analyses estimate adjusted longitudinal associations and an ordered coefficient product rather than causal effects or a causally identified mediation process (Cole and Maxwell, 2003; Maxwell and Cole, 2007; Cameron and Miller, 2015).

The predictive comparison used separate pooled ordinary least-squares models without community fixed effects, so prediction in held-out communities did not require estimating unobserved community-specific intercepts. Both models used the same 1,006 participants with complete data on the outcome and all predictors in the focal prediction model. The baseline model predicted Wave 4 SI from Wave 3 SI, age, the woman indicator, marginalized identity, and community tenure in months. The focal model added Wave 3 FB, community PG, and the marginalized identity × PG interaction. Before splitting the data, PG for this predictive comparison was standardized using the mean and population standard deviation of its assigned values across the 1,200 baseline records (4.007 and 0.719, respectively; denominator N). Community PG values were computed from Wave 1 responses; no Wave 4 outcomes were used to construct these predictors. The 60 community identifiers were sorted and assigned cyclically to five folds, with 12 communities per fold (Roberts et al., 2017). Each pair of models was fitted on four folds and applied to the remaining fold, yielding test-set sizes of 198, 208, 199, 205, and 196. The reported *R*^2^ was calculated once from all pooled out-of-fold predictions as 1 − Σ(*y* − *ŷ*)^2^/Σ(*y* − *ȳ*)^2^, where *ȳ* is the mean of all 1,006 observed Wave 4 SI scores; fold-specific *R*^2^ values were not averaged. This diagnostic evaluates prediction for held-out communities with baseline PG information available and does not validate the fixed-effects coefficients directly.

## 4 Results

### 4.1 Panel profile and missingness

The longitudinal sample comprised 1,200 participants at Wave 1, of whom 1,147 participated at Wave 2, 1,090 at Wave 3, and 1,012 at Wave 4. This corresponds to retention rates of 95.6%, 90.8%, and 84.3% relative to the baseline sample, respectively. Across the four measurement occasions, 8,724 item responses were missing as a result of participant attrition and item nonresponse. Baseline participant characteristics, community structure, panel retention, and missingness are summarized in Table 4.

### 4.2 Confirmatory factor structure, reliability, and convergent validity

The hypothesized five-factor model showed acceptable fit at each measurement occasion: Wave 1, MLR *χ*^2^(160) = 603.88, CFI = 0.949, TLI = 0.939, RMSEA = 0.059, 90% CI [0.054, 0.064], SRMR = 0.026, *N* = 807; Wave 2, MLR *χ*^2^(160) = 642.38, CFI = 0.942, TLI = 0.931, RMSEA = 0.063, 90% CI [0.058, 0.068], SRMR = 0.029, *N* = 753; Wave 3, MLR *χ*^2^(160) = 620.04, CFI = 0.943, TLI = 0.932, RMSEA = 0.061, 90% CI [0.056, 0.066], SRMR = 0.030, *N* = 760; and Wave 4, MLR *χ*^2^(160) = 599.97, CFI = 0.939, TLI = 0.928, RMSEA = 0.062, 90% CI [0.057, 0.068], SRMR = 0.030, *N* = 700, with the wave-specific fit indices and complete-item sample sizes reported in Table 5. The retained five-factor structure fit substantially better than the alternative structures: across waves, models combining AIM with DII or FB with SI yielded CFI values of 0.789–0.835, TLI values of 0.756–0.809, RMSEA values of 0.104–0.116, and SRMR values of 0.060–0.084, whereas the single-factor models showed poorer fit, with CFI values of 0.271–0.353, TLI values of 0.185–0.277, RMSEA values of 0.202–0.210, and SRMR values of 0.163–0.186, supporting the empirical differentiation of the five distinct but correlated focal constructs. Measurement quality was further supported by strong internal consistency, with coefficient alpha ranging from 0.866 to 0.878 and composite reliability from 0.863 to 0.880, all exceeding 0.86, while convergent validity was supported by average variance extracted values ranging from 0.611 to 0.648 and positive standardized factor loadings ranging from 0.742 to 0.856. No construct or measurement occasion showed a markedly weak loading pattern, and the close correspondence between coefficient alpha and composite reliability indicated that the reliability pattern was not dependent on a single method of estimating internal consistency. Taken together, the model fit comparisons, reliability coefficients, average variance extracted values, and standardized factor loadings indicate that the items assigned to AIM, DII, FB, SI, and PG showed coherent measurement within their intended constructs across all four waves, with the complete construct-specific results reported in Table 6.

**Table 5:** Confirmatory factor analysis fit by wave.

Wave	*N*	MLR *χ*^2^(*df*)	CFI	TLI	RMSEA [90% CI]	SRMR
1	807	603.88 (160)	0.949	0.939	0.059 [0.054, 0.064]	0.026
2	753	642.38 (160)	0.942	0.931	0.063 [0.058, 0.068]	0.029
3	760	620.04 (160)	0.943	0.932	0.061 [0.056, 0.066]	0.030
4	700	599.97 (160)	0.939	0.928	0.062 [0.057, 0.068]	0.030

MLR, robust maximum likelihood; CFI, comparative fit index; TLI, Tucker–Lewis index; RMSEA, root mean square error of approximation; SRMR, standardized root mean square residual. N is the number of cases with complete data on all 20 focal items at the corresponding wave.

**Table 6:** Reliability, convergent validity, and loading ranges by construct and wave.

Construct	Wave	Alpha	CR	AVE	*λ* min	*λ* max
AIM	1	0.873	0.878	0.644	0.753	0.839
DII	1	0.875	0.869	0.624	0.755	0.823
FB	1	0.874	0.872	0.631	0.757	0.829
SI	1	0.870	0.868	0.622	0.752	0.816
PG	1	0.869	0.870	0.627	0.777	0.813
AIM	2	0.878	0.880	0.648	0.766	0.841
DII	2	0.877	0.874	0.634	0.760	0.841
FB	2	0.877	0.875	0.637	0.774	0.817
SI	2	0.872	0.867	0.621	0.761	0.817
PG	2	0.869	0.872	0.631	0.767	0.816
AIM	3	0.871	0.874	0.634	0.769	0.825
DII	3	0.875	0.878	0.643	0.749	0.849
FB	3	0.870	0.868	0.623	0.755	0.821
SI	3	0.866	0.865	0.615	0.768	0.798
PG	3	0.869	0.865	0.616	0.742	0.830
AIM	4	0.866	0.863	0.611	0.747	0.822
DII	4	0.868	0.863	0.612	0.751	0.824
FB	4	0.869	0.865	0.617	0.779	0.795
SI	4	0.873	0.874	0.634	0.759	0.826
PG	4	0.870	0.871	0.628	0.754	0.856

CR, composite reliability calculated from standardized CFA loadings; AVE, average variance extracted; *λ*, standardized factor loading; AIM, algorithmic identity mirroring; DII, digital identity integration; FB, fan belonging; SI, perceived social inclusion; PG, participatory algorithmic governance.

### 4.3 Discriminant validity

Discriminant validity was supported across the five constructs and all four measurement occasions. All heterotrait–monotrait ratios were below 0.85, ranging from 0.026 to 0.516. The largest ratio was observed between fan belonging and perceived social inclusion at Wave 1 (HTMT = 0.516), followed by the same pair at Wave 2 (HTMT = 0.514) and algorithmic identity mirroring with digital identity integration at Wave 2 (HTMT = 0.505). Although these pairs represent theoretically adjacent constructs, their HTMT values remained well below the 0.85 criterion, indicating that they were empirically distinguishable. This pattern is consistent with the conceptual distinctions developed in Tables 1, 2. Algorithmic identity mirroring and digital identity integration remained distinguishable as perceived algorithmic recognition and the integration of identity-relevant experiences into the digital self, while fan belonging and perceived social inclusion remained distinguishable as relational membership and perceived access to valued participation and recognition. Participatory algorithmic governance showed low HTMT ratios with the individual-level psychological constructs across all four waves, ranging from 0.026 to 0.192. Taken together, these results indicate that AIM, DII, FB, SI, and PG represent related but empirically distinguishable constructs within the proposed framework. Complete HTMT results for all construct pairs and measurement occasions are reported in Table 7.

**Table 7:** Heterotrait-monotrait ratios by construct pair and wave.

Pair	Wave 1	Wave 2	Wave 3	Wave 4
AIM-DII	0.431	0.505	0.425	0.415
AIM-FB	0.241	0.230	0.198	0.110
AIM-PG	0.154	0.192	0.156	0.135
AIM-SI	0.293	0.205	0.146	0.115
DII-FB	0.318	0.349	0.293	0.266
DII-PG	0.026	0.071	0.036	0.050
DII-SI	0.331	0.215	0.204	0.135
FB-PG	0.032	0.046	0.052	0.034
FB-SI	0.516	0.514	0.486	0.445
SI-PG	0.039	0.042	0.068	0.044

HTMT, heterotrait-monotrait ratio; AIM, algorithmic identity mirroring; DII, digital identity integration; FB, fan belonging; SI, perceived social inclusion; PG, participatory algorithmic governance.

### 4.4 Descriptive statistics and correlations

Descriptive statistics and pairwise correlations are presented in Table 8. The strongest zero-order associations among the temporally adjacent focal constructs were observed between algorithmic identity mirroring at Wave 1 and digital identity integration at Wave 2, *r* = 0.442, and between fan belonging at Wave 3 and perceived social inclusion at Wave 4, *r* = 0.440. Digital identity integration at Wave 2 was also positively correlated with fan belonging at Wave 3, *r* = 0.352. Associations between constructs separated by longer intervals were positive but smaller: AIM at Wave 1 correlated with FB at Wave 3 at *r* = 0.256 and with SI at Wave 4 at *r* = 0.148, while DII at Wave 2 correlated with SI at Wave 4 at *r* = 0.227. The absolute zero-order correlations between PG climate and the individual-level focal constructs did not exceed 0.089. Overall, the correlation pattern was consistent with the proposed temporal ordering while leaving substantial variance among the constructs, and the adjusted longitudinal models reported below provide the formal tests of the hypotheses.

**Table 8:** Descriptive statistics and pairwise correlations.

Variable	*N*	*M*	SD	1	2	3	4	5
1 AIM W1	1,199	4.019	1.035	–	–	–	–	–
2 DII W2	1,146	4.004	1.046	0.442	–	–	–	–
3 FB W3	1,089	3.979	1.043	0.256	0.352	–	–	–
4 SI W4	1,009	4.016	1.036	0.148	0.227	0.440	–	–
5 PG climate	1,200	4.007	0.720	0.089	0.021	0.000	0.020	–

AIM, algorithmic identity mirroring; DII, digital identity integration; FB, fan belonging; SI, perceived social inclusion; PG, participatory algorithmic governance; W, wave. Correlations use pairwise available cases. PG climate is reported on its original scale. Its *N*, mean, standard deviation, and correlations summarize community means assigned to the 1,200 participant records, not 1,200 independent community observations. A standardized version was used in the interaction models, as specified in Section 3.4.

### 4.5 Governance aggregation

The aggregation diagnostics supported representing Wave 1 participatory algorithmic governance as a shared community-level climate. Across the 60 digital sports communities, an average of 19.95 valid participant responses contributed to each community estimate. ICC(1) was 0.469, indicating substantial between-community variation in individual assessments of participatory governance, while ICC(2) was 0.946, indicating high reliability of the aggregated community means. Median agreement within communities was 0.863, providing additional evidence that participants within the same community showed meaningful agreement in their perceptions of governance. Together, these results supported aggregation of individual PG assessments to the community level and provided the empirical basis for its use as a community-level moderator in the subsequent models (Table 9). Across the 60 unique community means, the sample standard deviation of PG climate was 0.726. Table 8 instead summarizes the assigned community means at the participant-record level (SD = 0.720), consistent with the scaling used in the primary interaction models.

**Table 9:** Community-level support for aggregating participatory algorithmic governance.

Metric	Value
Communities	60
Mean valid members per community	19.95
ICC(1)	0.469
ICC(2)	0.946
Median within-group agreement	0.863
PG sample standard deviation across 60 community means	0.726

ICC, intraclass correlation; PG, participatory algorithmic governance. Values concern participant assessments within 60 communities. The PG standard deviation is computed across the 60 unique community means using a sample denominator of 59; it is distinct from the participant-record-level SD in Table 8.

### 4.6 Primary lagged associations

All three adjusted lagged associations were positive and statistically significant. At mean governance climate (PGz = 0), within-community-centered algorithmic identity mirroring at Wave 1 was positively associated with digital identity integration at Wave 2, *B* = 0.284, SE = 0.031, *t*(59) = 9.20, *p* < 0.001, 95% CI [0.222, 0.346], supporting H1. Digital identity integration at Wave 2 was positively associated with fan belonging at Wave 3, *B* = 0.220, SE = 0.029, *t*(59) = 7.70, *p* < 0.001, 95% CI [0.163, 0.277], supporting H2. Fan belonging at Wave 3 was positively associated with perceived social inclusion at Wave 4, *B* = 0.204, SE = 0.029, *t*(59) = 6.97, *p* < 0.001, 95% CI [0.145, 0.263], supporting H3. These associations remained evident after adjustment for the prior-wave level of the corresponding outcome, participant characteristics, and community fixed effects. The results provide longitudinal evidence consistent with the three prospective associations specified in the proposed sequence, while the observational design and significant reverse-order diagnostics do not establish a unique causal direction. Full model estimates for the primary paths are reported in Table 10.

**Table 10:** Community fixed-effects estimates for the primary longitudinal associations.

Hyp.	Predictor	*B*	SE	*t*	*p*	95% CI	*N*
H1	AIMwc	0.284	0.031	9.20	<0.001	[0.222, 0.346]	1,143
H2	DII W2	0.220	0.029	7.70	<0.001	[0.163, 0.277]	1,086
H3	FB W3	0.204	0.029	6.97	<0.001	[0.145, 0.263]	1,006

B, unstandardized regression coefficient; SE, standard error; CI, confidence interval; AIM, algorithmic identity mirroring; DII, digital identity integration; FB, fan belonging; SI, perceived social inclusion; wc, centered within community; W, wave. Standard errors are clustered by community; tests use *t*(59). The H1 coefficient is evaluated at mean governance climate (PGz = 0) in the model containing the AIM × PGz interaction.

### 4.7 Ordered serial association

At mean governance climate (PGz = 0), the ordered product of the three adjusted lagged coefficients from the primary models was 0.0127. Community-level bootstrap estimation with 2,000 replications produced a bootstrap standard error of 0.0026 and a 95% confidence interval from 0.0080 to 0.0182. Because the confidence interval excluded zero, the result supported H4 and was consistent with the proposed ordered sequence, although the estimate was small and does not rule out reciprocal associations. Communities rather than individual participants were resampled during the bootstrap procedure to preserve the clustered structure of the longitudinal data. The complete estimate is reported in Table 11.

**Table 11:** Community bootstrap estimate of the ordered serial association.

Effect	Estimate	Bootstrap SE	95% CI	Replications	Decision
AIM W1 to DII W2 to FB W3 to SI W4	0.0127	0.0026	[0.0080, 0.0182]	2,000	Supported

AIM, algorithmic identity mirroring; DII, digital identity integration; FB, fan belonging; SI, perceived social inclusion. Communities, rather than individual participants, were resampled. The product uses the primary models, including their specified interaction terms, and is evaluated at PGz = 0 because the first-stage AIM association depends on PG.

### 4.8 Cross-level boundaries and marginalized identity

The cross-level interaction between algorithmic identity mirroring and participatory algorithmic governance was positive and statistically significant, *B* = 0.059, SE = 0.028, *t*(59) = 2.10, *p* = 0.040, 95% CI [0.003, 0.116], supporting H5. The estimate was modest and its lower confidence bound was close to zero, so this interaction should be interpreted cautiously. At mean governance climate (PGz = 0), marginalized identity was negatively associated with Wave 4 perceived social inclusion, *B* = −0.228, SE = 0.055, *t*(59) = −4.18, *p* < 0.001, 95% CI [−0.337, −0.119]. However, the interaction between marginalized identity and participatory algorithmic governance was not statistically significant, *B* = 0.005, SE = 0.070, *t*(59) = 0.07, *p* = 0.944, 95% CI [−0.135, 0.145]. The confidence interval included zero and the estimated interaction was small, providing no support for H6. Thus, participatory governance modestly moderated the association between algorithmic identity mirroring and subsequent digital identity integration, but the data did not support the hypothesized buffering role of participatory governance in the association between marginalized identity and later perceived social inclusion. The corresponding estimates are reported in Table 12.

**Table 12:** Cross-level moderation estimates and marginalized-identity association.

Hyp.	Effect	*B*	SE	*t*	*p*	95% CI	Decision
H5	AIM by PG	0.059	0.028	2.10	0.040	[0.003, 0.116]	Supported
Marginalized	Marginalized identity	−0.228	0.055	−4.18	<0.001	[−0.337, −0.119]	Negative association
H6	Marginalized identity by PG	0.005	0.070	0.07	0.944	[−0.135, 0.145]	Unsupported

*B*, unstandardized regression coefficient; SE, standard error; CI, confidence interval; AIM, algorithmic identity mirroring; PG, participatory algorithmic governance. Standard errors are clustered by community; tests use *t*(59). PGz is standardized as described in Section 3.4. The marginalized-identity coefficient is evaluated at PGz = 0; the interaction coefficients quantify change in the corresponding association per one-unit increase in PGz.

### 4.9 Alternative ordering and robustness checks

The diagnostic analyses retained the primary positive associations but also indicated that reciprocal dynamics remain plausible. Wave 1 digital identity integration was positively associated with Wave 2 algorithmic identity mirroring, *B* = 0.108, SE = 0.030, *p* < 0.001. Wave 2 fan belonging was positively associated with Wave 3 digital identity integration, *B* = 0.082, SE = 0.029, *p* = 0.006. Wave 3 perceived social inclusion was also positively associated with Wave 4 fan belonging, *B* = 0.114, SE = 0.032, *p* < 0.001. Each reverse coefficient was smaller than its corresponding focal coefficient, but the significant reverse associations preclude a strictly one-way interpretation of the proposed sequence. The complete four-item estimate for H1 was 0.291 (*N* = 889), closely approximating the primary estimate of 0.284 and indicating that the first-stage association was not materially altered when complete responses were required for AIM at Wave 1 and DII at Waves 1 and 2. In the separate pooled prediction models without community fixed effects, the pooled out-of-fold *R*^2^ was 0.410 for the focal model and 0.375 for the baseline model (*N* = 1,006), using the community-blocked procedure specified in Section 3.5. The complete-item check supports the stability of the H1 estimate under the stricter scoring rule, while the predictive comparison shows a modest improvement for the separate focal prediction model. Neither result establishes a unique causal direction or directly validates all primary coefficients (Table 13).

**Table 13:** Alternative temporal ordering, complete-item sensitivity, and predictive checks.

Check	Estimate	SE	*p*
Reverse: DII W1 to AIM W2	0.108	0.030	<0.001
Reverse: FB W2 to DII W3	0.082	0.029	0.006
Reverse: SI W3 to FB W4	0.114	0.032	<0.001
Complete-four-item H1 (*N* = 889)	0.291	–	–
Pooled out-of-fold *R*^2^, focal model	0.410	–	–
Pooled out-of-fold *R*^2^, baseline model	0.375	–	–

Reverse-order models use community-clustered SEs and *t*(59). The complete-four-item H1 sensitivity requires complete item responses for AIM at Wave 1 and DII at Waves 1 and 2. *R*^2^, coefficient of determination. Diagnostic comparisons do not establish causal direction. Prediction models exclude community fixed effects and use a common sample of 1,006 participants. The baseline includes SI W3, age, woman, marginalized identity, and tenure; the focal model additionally includes FB W3, PG, and marginalized identity × PG. Reported *R*^2^ pools all out-of-fold predictions; see Section 3.5 for the fold allocation and scaling.

### 4.10 Hypothesis summary

Table 14 summarizes the hypothesis tests. H1 through H5 met the prespecified support criterion, although the H5 interaction was modest and less precisely estimated than the other supported associations. H6 was unsupported. Marginalized identity was negatively associated with later perceived social inclusion, but participatory algorithmic governance did not significantly attenuate this association.

**Table 14:** Consolidated hypothesis summary.

Hyp.	Effect	*B*	95% CI	*p*	Decision
H1	AIM W1 to DII W2	0.284	[0.222, 0.346]	<0.001	Supported
H2	DII W2 to FB W3	0.220	[0.163, 0.277]	<0.001	Supported
H3	FB W3 to SI W4	0.204	[0.145, 0.263]	<0.001	Supported
H4	AIM W1 to DII W2 to FB W3 to SI W4	0.013	[0.008, 0.018]	–	Supported
H5	AIM by PG	0.059	[0.003, 0.116]	0.040	Supported
Additional association	Marginalized identity	−0.228	[−0.337, −0.119]	<0.001	Negative association
H6	Marginalized identity by PG	0.005	[−0.135, 0.145]	0.944	Unsupported

*B*, unstandardized regression coefficient; CI, confidence interval; AIM, algorithmic identity mirroring; DII, digital identity integration; FB, fan belonging; SI, perceived social inclusion; PG, participatory algorithmic governance; W, wave. H4 represents the ordered coefficient product and uses 2,000 community-level bootstrap replications. All other hypothesis tests use standard errors clustered by community and *t* inference with 59 degrees of freedom. H1, H4, and the additional marginalized-identity association are evaluated at mean governance climate (PGz = 0).

## 5 Discussion

### 5.1 Integration and belonging as distinct transitions

The association between earlier algorithmic identity mirroring and subsequent digital identity integration is particularly informative because it distinguishes perceived algorithmic recognition from more familiar evaluations of digital systems. Personalization concerns whether content corresponds with user preferences, algorithmic trust concerns reliance on the system, and self-congruity concerns correspondence between the self and an object or brand. Algorithmic identity mirroring instead captures the perception that an intelligent environment recognizes and reflects meaningful aspects of the self. The findings are therefore consistent with the proposition that perceived algorithmic recognition can become relevant to the organization of digital identity rather than functioning only as an assessment of recommendation relevance. This interpretation extends research on algorithmic imaginaries and folk theories by suggesting that users may respond psychologically not only to what computational systems do, but also to what they believe those systems notice and reflect about them (Bucher, 2017; DeVito et al., 2018).

The findings further reinforce the distinction between self-organization and relational confirmation within the proposed framework. Digital identity integration concerns the coherence of fandom experienced through digital platforms with the wider digital self, whereas fan belonging concerns acceptance, membership, and relational embeddedness within a focal community. Perceived social inclusion extends beyond both by capturing perceived access to valued participation and recognition. These distinctions matter because strong team identification does not necessarily imply community acceptance, and a sense of belonging does not necessarily imply equal access to participation. Table 2 formalizes these conceptual boundaries and helps prevent theoretically adjacent constructs from being treated as interchangeable.

The positive ordered association at mean governance climate is consistent with treating identity integration, belonging, and inclusion as distinct and temporally ordered processes rather than interchangeable constructs. The significant reverse-order diagnostics, however, indicate that reciprocal relations remain plausible and that the proposed sequence should not be interpreted as an exclusive direction of influence. This interpretation is compatible with research on social identity, psychological sense of community, and brand communities (McMillan and Chavis, 1986; Muniz and O’Guinn, 2001; McAlexander et al., 2002; Algesheimer et al., 2005). Future research could examine these reciprocal possibilities and whether the prospective associations are also reflected in behavioral participation, bridging social ties, and network diversity.

### 5.2 Procedural legitimacy is not substantive inclusion

The contrast between H5 and H6 identifies an important boundary in the role of participatory algorithmic governance. Higher participatory governance was associated with a modest strengthening of the prospective association between algorithmic identity mirroring and subsequent digital identity integration, consistent with the proposition that voice, contestability, and opportunities for feedback may make perceived recognition more accountable and meaningful to community members. Because the interaction was small and its confidence interval approached zero, this result requires replication. The same governance climate did not significantly moderate the negative association between marginalized identity and later perceived social inclusion. This contrast suggests that procedural legitimacy and substantive inclusion should not be treated as equivalent outcomes. Mechanisms that provide voice, feedback, or opportunities for appeal may shape how users interpret algorithmic recognition without necessarily altering the broader conditions associated with participation and inclusion. In particular, improved procedures cannot be assumed to change representation, safety, exposure, accessibility, or the resources required for meaningful participation (Binns, 2018; Benjamin, 2019; Noble, 2018).

The negative association between marginalized identity and later perceived social inclusion further indicates that favorable governance perceptions do not necessarily correspond with the absence of identity-related disparities. At the same time, the unsupported interaction in H6 should not be interpreted as evidence that participatory governance can never reduce such disparities. Rather, the present data did not provide evidence that community PG attenuated the observed negative association within this sample and study design. Because the marginalized identity measure captured broad self-identified marginalization rather than specific identity categories, this finding should be interpreted at that aggregate level rather than attributed to any particular group or generalized to marginalized populations as a whole. Future research should measure specific identity categories directly, examine heterogeneity across them, and investigate whether particular governance arrangements, such as representation, accessibility, safety mechanisms, or meaningful opportunities for contestation, are differentially associated with substantive inclusion.

### 5.3 Concrete empirical tests

A first experiment could use a 2 × 2 factorial design that independently manipulates algorithmic identity mirroring and participatory governance. Participants could receive either sports recommendations designed to reflect identity-relevant preferences or more generic recommendations, together with either meaningful opportunities to question and contest recommendations or an interface without such opportunities. Preregistered outcomes could include digital identity integration immediately after exposure and fan belonging following a structured community interaction. Manipulation checks should distinguish perceived recognition from content relevance, while behavioral measures could complement questionnaire responses. Such a design would provide a more direct test of H1 and H5 by experimentally separating the experience of algorithmic recognition from ordinary personalization and by manipulating the governance conditions under which that recognition is interpreted.

A second study could extend the present longitudinal evidence through a field intervention conducted across multiple digital fan communities. Communities could receive governance features such as transparent explanations of curation practices, representative participation in rule development, accessible appeal channels, and supports addressing safety or accessibility. Repeated surveys could be combined with consented behavioral logs, moderation records, and appeal outcomes to distinguish procedural participation from substantive inclusion. The design could also recruit sufficient participants from marginalized identity groups to permit preregistered subgroup comparisons and examine whether changes in perceived governance correspond with changes in actual opportunities for participation. Such a study would provide a stronger test of the unresolved relationship between participatory procedures and substantive inclusion identified by the contrast between H5 and H6.

### 5.4 Limitations

Several limitations should qualify the interpretation of the findings. First, although the four-wave longitudinal design establishes temporal separation among the focal constructs and adjusts for prior levels of the corresponding outcomes, the study remains observational. Community fixed effects account for time-invariant differences across communities, but they cannot eliminate unmeasured confounding that varies over time or across individuals. The lagged associations and ordered coefficient product should therefore not be interpreted as causal effects or as a causally identified mediation process. Experimental and intervention studies are needed to determine whether changes in algorithmic identity mirroring produce subsequent changes in identity integration, belonging, or inclusion. Second, the measures rely on participant reports. Perceived algorithmic identity mirroring captures how users interpret curated content and does not establish what recommendation systems actually inferred, selected, or optimized. Future studies could combine questionnaire measures with consented behavioral records or platform data to examine correspondence between perceived recognition and actual patterns of algorithmic curation.

Third, the newly operationalized constructs require further validation beyond the present sample. Although the measures showed strong internal consistency, convergent validity, and discriminant validity across the four measurement occasions, the global fit indices were acceptable rather than uniformly excellent, and independent replication would strengthen confidence in their measurement properties. Longitudinal measurement invariance was not evaluated and should be examined in future research. Fourth, participants were recruited in China through multiple online and community-based channels, which broadens the recruitment context but does not establish representativeness of all digital sports community users or users in other cultural settings. Replication across different populations, sports contexts, and platform environments would help establish the generalizability of the findings. Finally, the marginalized identity indicator was a single binary self-identification item that did not distinguish specific identity categories, so the associated findings reflect broad self-identified marginalization and should not be attributed to any particular group or generalized to marginalized populations as a whole. Future research with sufficiently large and diverse samples could examine heterogeneity across specific identity groups and determine whether different forms of participatory governance are associated with different inclusion outcomes.

## 6 Conclusion

This study develops and evaluates a bounded psychological framework of algorithmic fandom using a four-wave, multilevel longitudinal survey of digital sports community participants in China. The results support the proposed longitudinal associations while distinguishing the focal constructs as related but conceptually and empirically distinct. Significant reverse-order diagnostics indicate that reciprocal relations remain plausible, so the proposed sequence should not be interpreted as a unique causal direction. Participatory algorithmic governance was associated with a modest strengthening of the prospective association between perceived algorithmic recognition and subsequent identity integration, but the data did not support its hypothesized buffering role in the negative association between marginalized identity and later perceived social inclusion. This contrast highlights the distinction between procedural legitimacy and substantive inclusion: opportunities for voice, feedback, and contestation may strengthen the psychological significance of algorithmic recognition without necessarily reducing identity-related disparities in participation and inclusion. Platform governance initiatives may therefore benefit from considering participatory mechanisms alongside representation, safety, accessibility, and meaningful opportunities for participation.

## Data Availability

The raw data supporting the conclusions of this article will be made available by the authors, without undue reservation.
