# Supplementary material for: Algorithmic fandom in the age of AI: a four-wave multilevel longitudinal study of identity mirroring, fan belonging, and social inclusion
Source: Front Psychol. 2026 Oct 19;17:1955806. doi: 10.3389/fpsyg.2026.1955806 (PMC13724409; doi:10.3389/fpsyg.2026.1955806)
Supplement: Supplementary file 1 [file Supplementary_file_1.docx]

# Supplementary Table S1

## Questionnaire items, English translations, response options, and scoring procedures

The questionnaire was administered in Chinese through Wenjuanxing. Participants first identified the digital sports platform or related community that they used most frequently from the five platform contexts included in the study. This selected platform served as the focal reference for all subsequent items concerning algorithmic identity mirroring, digital identity integration, fan belonging, perceived social inclusion, and participatory algorithmic governance.

Unless otherwise indicated, the 20 focal construct items were rated on a seven-point scale ranging from **1 = strongly disagree** to **7 = strongly agree**. Each focal construct contained four items. The fourth item of each construct was negatively keyed and reverse scored as **8 minus the original response** before calculation of the scale score.

### A. Focal digital sports platform selection

| **Variable** | **Original Chinese wording** | **English translation** | **Response options** | **Use in the study** |
| --- | --- | --- | --- | --- |
| Focal digital sports platform | **在以下体育数字平台或相关线上社群中，您最常使用或参与的是哪一个？请选择一项。后续问题均以您选择的平台及其相关社群为回答对象。** | **Which of the following digital sports platforms or related online communities do you use or participate in most frequently? Please select one. All subsequent questions refer to the platform and its related community that you select.** | 1. NBA App / NBA digital community; 2. ESPN; 3. Nike App / Nike digital community; 4. adidas App / adidas digital community; 5. Strava | The selected platform defined the focal digital sports platform and related community for subsequent questionnaire items. |

**Instruction presented before the focal construct items**

**Chinese:**
以下问题中的“平台”和“社群”均指您刚才选择的体育数字平台及其相关线上社群。请根据您实际使用该平台和参与相关社群的体验作答。

**English translation:**
In the following items, “the platform” and “the community” refer to the digital sports platform and its related online community that you selected above. Please answer based on your actual experiences of using the platform and participating in the related community.

### B. Algorithmic identity mirroring

| **Item** | **Original Chinese wording** | **English translation** | **Response** | **Scoring** |
| --- | --- | --- | --- | --- |
| AIM1 | 平台向我推荐的体育内容能够反映我作为体育爱好者的重要特征。 | The sports content recommended to me by the platform reflects important aspects of who I am as a sports fan. | 1 to 7 | Direct |
| AIM2 | 平台推荐的内容能够体现我的体育偏好、价值观或群体归属。 | The content recommended by the platform reflects my sports preferences, values, or group affiliations. | 1 to 7 | Direct |
| AIM3 | 从平台为我呈现的内容中，我能感受到它似乎理解我是怎样的体育爱好者。 | From the content presented to me, I feel that the platform seems to understand what kind of sports fan I am. | 1 to 7 | Direct |
| AIM4 | 平台向我推荐的体育内容很少能反映我是谁。 | The sports content recommended to me by the platform rarely reflects who I am. | 1 to 7 | Reverse |

### C. Digital identity integration

| **Item** | **Original Chinese wording** | **English translation** | **Response** | **Scoring** |
| --- | --- | --- | --- | --- |
| DII1 | 我在该体育数字平台上的粉丝身份与我整体的网络自我是一致的。 | My fan identity on this digital sports platform is consistent with my broader digital self. | 1 to 7 | Direct |
| DII2 | 我在该平台上的粉丝经历已经成为我网络身份中有意义的一部分。 | My experiences as a fan on this platform have become a meaningful part of my digital identity. | 1 to 7 | Direct |
| DII3 | 我能够将自己在该平台上的粉丝经历与整体网络自我联系起来。 | I can connect my experiences as a fan on this platform with my broader digital self. | 1 to 7 | Direct |
| DII4 | 我在该平台上的粉丝身份与我整体的网络自我是相互割裂的。 | My fan identity on this platform feels disconnected from my broader digital self. | 1 to 7 | Reverse |

### D. Fan belonging

| **Item** | **Original Chinese wording** | **English translation** | **Response** | **Scoring** |
| --- | --- | --- | --- | --- |
| FB1 | 我觉得自己是这个体育粉丝社群的一员。 | I feel that I am a member of this sports fan community. | 1 to 7 | Direct |
| FB2 | 在这个体育粉丝社群中，我感到自己被其他成员接纳。 | I feel accepted by other members of this sports fan community. | 1 to 7 | Direct |
| FB3 | 我与这个体育粉丝社群中的其他成员有一种联系感。 | I feel a sense of connection with other members of this sports fan community. | 1 to 7 | Direct |
| FB4 | 即使参与这个体育粉丝社群，我仍然觉得自己像一个局外人。 | Even when participating in this sports fan community, I still feel like an outsider. | 1 to 7 | Reverse |

### E. Perceived social inclusion

| **Item** | **Original Chinese wording** | **English translation** | **Response** | **Scoring** |
| --- | --- | --- | --- | --- |
| SI1 | 在这个数字体育粉丝社群中，我觉得自己有机会参与对我有意义的活动和交流。 | In this digital sports fan community, I feel that I have opportunities to participate in activities and interactions that are meaningful to me. | 1 to 7 | Direct |
| SI2 | 在这个数字体育粉丝社群中，我觉得自己的参与能够得到认可。 | In this digital sports fan community, I feel that my participation is recognized. | 1 to 7 | Direct |
| SI3 | 在这个数字体育粉丝社群中，我能够获得对我有价值的社会互动和参与机会。 | In this digital sports fan community, I have access to social interactions and opportunities for participation that I value. | 1 to 7 | Direct |
| SI4 | 在这个数字体育粉丝社群中，我经常觉得自己被排除在有意义的参与之外。 | In this digital sports fan community, I often feel excluded from meaningful participation. | 1 to 7 | Reverse |

### F. Participatory algorithmic governance

| **Item** | **Original Chinese wording** | **English translation** | **Response** | **Scoring** |
| --- | --- | --- | --- | --- |
| PG1 | 在这个社群中，成员有机会对平台的内容推荐或相关规则提出意见。 | In this community, members have opportunities to provide input on the platform’s content recommendations or related rules. | 1 to 7 | Direct |
| PG2 | 当平台的内容推荐出现问题时，成员有明确的反馈或申诉渠道。 | When problems arise with the platform’s content recommendations, members have clear channels for feedback or appeal. | 1 to 7 | Direct |
| PG3 | 在与内容推荐有关的规则或程序中，不同成员的意见能够得到体现。 | The views of different members are represented in rules or procedures related to content recommendations. | 1 to 7 | Direct |
| PG4 | 对于平台如何推荐和管理内容，社群成员几乎没有表达意见或提出异议的机会。 | Community members have little opportunity to express their views or raise objections about how the platform recommends and manages content. | 1 to 7 | Reverse |

### G. Marginalized identity indicator

| **Variable** | **Original Chinese wording** | **English translation** | **Response options** | **Coding** |
| --- | --- | --- | --- | --- |
| Marginalized identity | **您是否认为自己属于边缘化或社会弱势身份群体？例如，因社会身份、经济或社会处境、身体状况或其他身份特征而可能在社会参与中处于不利位置的群体。** | **Do you consider yourself to belong to a marginalized or socially disadvantaged identity group? For example, this may include groups that may experience disadvantage in social participation because of social identity, socioeconomic circumstances, physical condition, or other identity characteristics.** | Yes / No | Yes = 1; No = 0 |

**Clarification.** The examples accompanying this item were intended only to clarify the meaning of marginalized or socially disadvantaged identity. Participants were not required to identify or select a particular identity category. The resulting variable therefore represents broad self-identified marginalization and does not permit inference regarding any specific identity subgroup. This is consistent with the interpretation used in the main manuscript.

### Response scale

For AIM, DII, FB, SI, and PG:

| **Value** | **Response** |
| --- | --- |
| 1 | Strongly disagree |
| 2 | Disagree |
| 3 | Somewhat disagree |
| 4 | Neither agree nor disagree |
| 5 | Somewhat agree |
| 6 | Agree |
| 7 | Strongly agree |

### Scoring

For each focal construct, Items 1 to 3 were scored directly. Item 4 was negatively keyed and reverse scored using:

**Reverse scored response = 8 minus the original response.**

A scale score was calculated when at least three of the four corresponding items were available, consistent with the procedure reported in the main manuscript.

### Note

AIM, algorithmic identity mirroring; DII, digital identity integration; FB, fan belonging; SI, perceived social inclusion; PG, participatory algorithmic governance. The focal digital sports platform selected by each participant served as the reference context for the subsequent construct items. The five focal constructs were assessed using four items each on a seven-point response scale. The fourth item of each construct was negatively keyed and reverse scored as 8 minus the original response. The marginalized identity indicator was assessed separately from the five focal constructs and coded 1 for yes and 0 for no.

### Supplementary Note S1. Measure development and administration

The questionnaire was administered in Chinese through Wenjuanxing. Participants first selected the digital sports platform or related online community that they used or participated in most frequently from the five platform contexts included in the study. The selected platform and its related community served as the focal reference for the subsequent questionnaire items. The five focal measures were developed to operationalize the constructs specified in the theoretical framework. Before data collection, two experts reviewed the construct definitions and item content for consistency with the intended conceptual domains. Both experts judged the proposed content to be consistent with the intended constructs, and no changes were made following review. The English translations in Supplementary Table S1 are provided for transparent reporting and replication and were not administered to participants. Measurement evidence concerning internal consistency, composite reliability, convergent validity, discriminant validity, and confirmatory factor structure is reported in the main manuscript. The marginalized identity indicator was analyzed separately from the five focal multi-item measures.
